# Supplementary material for: A randomized clinical trial to stimulate the cholinergic anti-inflammatory pathway in patients with moderate COVID-19-pneumonia using a slow-paced breathing technique
Source: Front Immunol. 2022 Oct 3;13:928979. doi: 10.3389/fimmu.2022.928979 (PMC9574246; doi:10.3389/fimmu.2022.928979)
Supplement: Supplementary file 1 [file DataSheet_1.pdf]

Supplemental Table 1 Figure 1: Response of HRV parameters (HF and LF) to slow-paced breathing

Phase	Variable	mean	sd	p25	p50	p75
**Rest pre Intervention**	**HF (ln[ms²])**	3.83	1.54	2.76	3.85	4.78
	**LF (ln[ms²])**	4.77	1.28	4.00	4.80	5.48
**Breathing Intervention**	**HF (ln[ms²])**	4.10	1.42	3.08	4.01	5.00
	**LF (ln[ms²])**	5.58	1.26	4.91	5.65	6.42
**Rest post Intervention**	**HF (ln[ms²])**	3.81	1.39	3.04	3.67	4.65
	**LF (ln[ms²])**	5.18	1.10	4.32	5.21	6.13

DOI: 10.3389/fimmu.2022.928979

Supplemental Figure 1: Response of HRV parameters (HF and LF) to slow-paced breathing

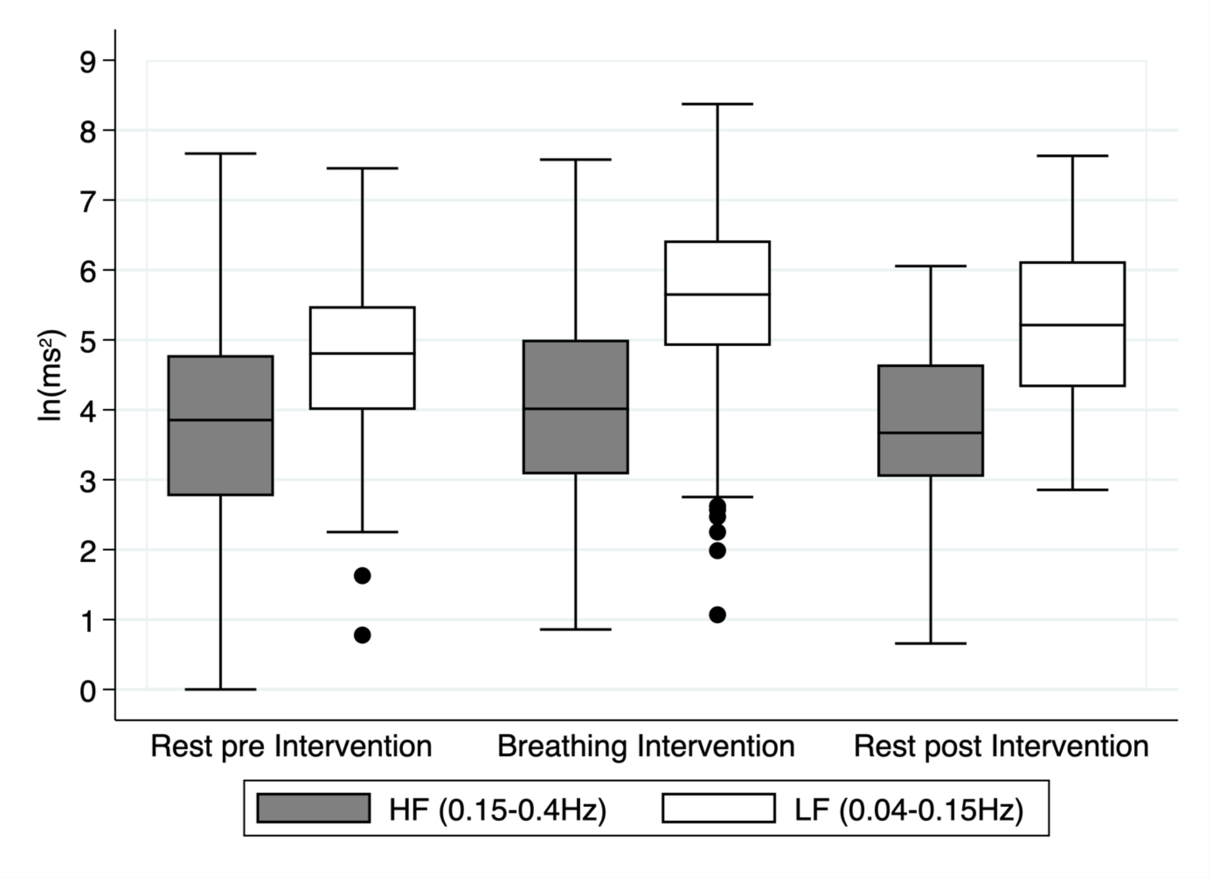

Legend:
Rest pre Intervention: Resting condition, free breathing, laid upright in bed
Breathing Intervention: Slow-paced breathing at 6 breaths/min (inhalation:exhalation = 4sec:6sec), position laid upright in bed
Rest post Intervention: Resting condition, free breathing, laid upright in bed
HF: log-transformed High Frequency power (ms^2) autoregressive
LF: log-transformed Low Frequency power (ms^2) autoregressive
Note: The adult standard breathing frequency of 12-18 breaths per minute (BPM; = 0.2-0.3Hz) is usually within the HF-Band (0.15-0.4Hz), which is considered to reflect primarily vagal activity. However, under paced breathing conditions at 6BPM (=0.1Hz), this peak is shifted towards the LF-Band (0.04-0.15Hz)

DOI: 10.3389/fimmu.2022.928979

BEAT COVID

UNIVERSITÄTS KLINIKUM ulm

Instruction Slow-Paced Breathing Exercise

Our breathing and heartbeat are closely related to each other. When we inhale, our heart beats faster. When we exhale, it beats slower. This connection is based on your autonomic nervous system. This system controls a number of other important body functions, such as your blood pressure and immune system. Your autonomic nervous system is guided by your subconscious, but you can specifically influence the activity of your autonomic nervous system through your breathing - and by doing that also influence your immune system in addition to your heartbeat.

The effects on your heart can be determined by means of measurements, e.g., the fluctuation of your pulse, the so-called heart rate variability. This measurement is done using a simple chest strap that records the electrical signal from the heart muscle. By taking blood samples we measure the influence on the immune system. We want to investigate to what extent breathing exercises can improve heart rate variability and inflammatory markers.

In order to achieve an effect, it is important that you do the breathing exercise often enough and for a long time. **You should practice three times a day for 20 minutes.** However, it is also important that you feel comfortable doing the exercise. No effect is achieved under excitement or tension. If you do the exercise correctly, you will always feel more relaxed. You may become tired or even fall asleep. This is proof that you have performed the exercise correctly.

If you feel uncomfortable during the exercise or your fingers start tingling, you get dizzy or show signs of headache and nausea then something is wrong and you should stop the exercise and contact the study staff. They will check with you whether you need to improve your technique or rather stop doing the exercise.

The BreathBall app will help you doing the exercise on your own (Setting 4:6 Inhale:Exhale ratio)

Brief instruction (by study personnel):

- When the breath ball gets bigger, you should inhale.
- When it gets smaller, you should exhale.
- Here you can start the exercise, here you can stop it, here you can adjust the speed.
- Once again, it is very important that you breathe calmly and in a relaxed manner. Do not overexert yourself. If you feel dizzy, try to breathe more shallowly.
- Now go ahead and try Breath Ball.
- Be sure to breathe into your belly. You can put one hand on your belly and one on your chest in order to get a better feeling for the exercise. The hand on your belly should move more. Like this: (show).
- While doing this, you can also imagine breathing in all the way down to your feet.
- Slightly pressing your lips together while exhaling (lip brake) can also help.

DOI: 10.3389/fimmu.2022.928979

BEAT COVID

UNIVERSITÄTS KLINIKUM ulm

Anleitung Atemübung

Ihre Atmung und Ihr Herzschlag sind eng miteinander gekoppelt. Wenn Sie einatmen, schlägt Ihr Herz schneller, wenn Sie ausatmen, schlägt es langsamer. Diese Kopplung erfolgt über Ihr autonomes Nervensystem. Dieses steuert noch eine Vielzahl an anderen, wichtigen Körperprozessen wie beispielsweise ihren Blutdruck und oder auch ihr Immunsystem. Das autonome Nervensystem arbeitet zumeist ohne Ihre bewusste Mitwirkung aber Sie können durch Ihre Atmung die Aktivität Ihres autonomen Nervensystems gezielt beeinflussen – und folglich auch neben ihren Herzschlag auch ihr Immunsystem beeinflussen.

Den Einfluss auf das Herz kann man leicht messen, indem man die Schwankungsbreite Ihres Pulses misst, die sogenannte Herzratenvariabilität. Diese Messung erfolgt über einen einfachen Brustgurt, der das elektrische Signal des Herzmuskels aufzeichnet. Den Einfluss auf das Immunsystem messen wir mittels Blutentnahmen. Wir wollen Untersuchen, in welchem Ausmaß die Atemübung die Herzratenvariabilität und die Entzündungsmarker verbessern können.

Es ist wichtig, dass Sie die Atemübung ausreichend oft und lange durchführen, um wirklich einen Effekt zu erreichen. Sie sollten diese dreimal am Tag für 20 Minuten durchführen. Es ist aber auch wichtig, dass Sie sich bei der Übung wohlfühlen; wenn Sie sich dabei anspannen und verkrampfen, wird kein Effekt eintreten. Wenn Sie die Übung richtig durchführen, fühlen Sie sich dabei immer entspannter und lockerer. Es kann sein, dass Sie müde werden oder sogar einschlafen. Dann machen Sie die Übung richtig.

Wenn Sie sich während der Übung unwohl fühlen und Sie folgendes spüren: Kribbeln in den Fingern, Schwindel, Übelkeit, Kopfweh; dann stimmt etwas nicht und Sie sollten die Übung abbrechen und die Studienmitarbeiterin kontaktieren. Sie wird dann zusammen mit Ihnen überprüfen, ob Sie diese Beschwerden durch eine Verbesserung Ihrer Technik beheben können oder ob Sie lieber die Übungen nicht mehr durchführen sollten.

Bei der Übung selbst hilft Ihnen die App *BreathBall*. Ich mache es Ihnen vor:

Wenn die Atemkugel größer wird, atmen Sie ein -

Wenn Sie kleiner wird, atmen Sie aus.

Hier können Sie die Übung starten, hier stoppen, hier können Sie die Geschwindigkeit verstellen.

Noch einmal: es ist ganz wichtig, dass Sie ruhig und entspannt atmen. Strengen Sie sich dabei nicht allzu sehr an. Wenn Ihnen schwindlig werden sollte, atmen Sie flacher.

Jetzt probieren Sie es: Atemkugel –

Achten Sie darauf, in den Bauch zu atmen. Sie können zur Hilfe eine Hand auf Ihren Bauch und eine auf Ihre Brust legen. Die auf dem Bauch sollte sich mehr bewegen. So: (vormachen)

Sie können sich dabei auch vorstellen, dass Sie bis zu Ihren Füßen einatmen.

Es kann auch helfen, die Lippen leicht zusammenzupressen beim Ausatmen (Lippenbremse).

DOI: 10.3389/fimmu.2022.928979

CONSORT 2010 checklist of information to include when reporting a randomised trial*

Section/Topic	Item No	Checklist item	Reported on page No
**Title and abstract**			
	1a	Identification as a randomised trial in the title	n.a.
	1b	Structured summary of trial design, methods, results, and conclusions (for specific guidance see CONSORT for abstracts)	2
**Introduction**			
Background and objectives	2a	Scientific background and explanation of rationale	4
	2b	Specific objectives or hypotheses	5
**Methods**			
Trial design	3a	Description of trial design (such as parallel, factorial) including allocation ratio	6
	3b	Important changes to methods after trial commencement (such as eligibility criteria), with reasons	n.a.
Participants	4a	Eligibility criteria for participants	6
	4b	Settings and locations where the data were collected	6
Interventions	5	The interventions for each group with sufficient details to allow replication, including how and when they were actually administered	6,7
Outcomes	6a	Completely defined pre-specified primary and secondary outcome measures, including how and when they were assessed	7/8
	6b	Any changes to trial outcomes after the trial commenced, with reasons	n.a.
Sample size	7a	How sample size was determined	8
	7b	When applicable, explanation of any interim analyses and stopping guidelines	8
Randomisation:			
Sequence generation	8a	Method used to generate the random allocation sequence	6/7
	8b	Type of randomisation; details of any restriction (such as blocking and block size)	7
Allocation concealment mechanism	9	Mechanism used to implement the random allocation sequence (such as sequentially numbered containers), describing any steps taken to conceal the sequence until interventions were assigned	7

DOI: 10.3389/fimmu.2022.928979

Implementation	10	Who generated the random allocation sequence, who enrolled participants, and who assigned participants to interventions	7
Blinding	11a	If done, who was blinded after assignment to interventions (for example, participants, care providers, those assessing outcomes) and how	n.a.
	11b	If relevant, description of the similarity of interventions	n.a.
Statistical methods	12a	Statistical methods used to compare groups for primary and secondary outcomes	8/9
	12b	Methods for additional analyses, such as subgroup analyses and adjusted analyses	8/9
**Results**			
Participant flow (a diagram is strongly recommended)	13a	For each group, the numbers of participants who were randomly assigned, received intended treatment, and were analysed for the primary outcome	Figure 1
	13b	For each group, losses and exclusions after randomisation, together with reasons	Figure 1
Recruitment	14a	Dates defining the periods of recruitment and follow-up	8
	14b	Why the trial ended or was stopped	9
Baseline data	15	A table showing baseline demographic and clinical characteristics for each group	Table 1
Numbers analysed	16	For each group, number of participants (denominator) included in each analysis and whether the analysis was by original assigned groups	10 / 11
Outcomes and estimation	17a	For each primary and secondary outcome, results for each group, and the estimated effect size and its precision (such as 95% confidence interval)	12
	17b	For binary outcomes, presentation of both absolute and relative effect sizes is recommended	n.a.
Ancillary analyses	18	Results of any other analyses performed, including subgroup analyses and adjusted analyses, distinguishing pre-specified from exploratory	13
Harms	19	All important harms or unintended effects in each group (for specific guidance see CONSORT for harms)	13
**Discussion**			
Limitations	20	Trial limitations, addressing sources of potential bias, imprecision, and, if relevant, multiplicity of analyses	12
Generalisability	21	Generalisability (external validity, applicability) of the trial findings	11/12
Interpretation	22	Interpretation consistent with results, balancing benefits and harms, and considering other relevant evidence	11/12
**Other information**			
Registration	23	Registration number and name of trial registry	5
Protocol	24	Where the full trial protocol can be accessed, if available	supplement
Funding	25	Sources of funding and other support (such as supply of drugs), role of funders	17

DOI: 10.3389/fimmu.2022.928979

*We strongly recommend reading this statement in conjunction with the CONSORT 2010 Explanation and Elaboration for important clarifications on all the items. If relevant, we also recommend reading CONSORT extensions for cluster randomised trials, non-inferiority and equivalence trials, non-pharmacological treatments, herbal interventions, and pragmatic trials. Additional extensions are forthcoming: for those and for up to date references relevant to this checklist, see www.consort-statement.org.

DOI: 10.3389/fimmu.2022.928979
